# Genome Sequence of Acinetobacter baumannii Strain A1, an Early Example of Antibiotic-Resistant Global Clone 1

**DOI:** 10.1128/genomeA.00032-15

**Published:** 2015-03-12

**Authors:** Kathryn E. Holt, Mohammad Hamidian, Johanna J. Kenyon, Matthew T. Wynn, Jane Hawkey, Derek Pickard, Ruth M. Hall

**Affiliations:** a Department of Biochemistry and Molecular Biology, Bio21 Molecular Science and Biotechnology Institute, University of Melbourne, Melbourne, Australia; b School of Molecular Bioscience, The University of Sydney, Sydney, Australia; c Wellcome Trust Sanger Institute, Hinxton, United Kingdom

## Abstract

Acinetobacter baumannii isolate A1 was recovered in the United Kingdom in 1982 and belongs to global clone 1 (GC1). Here, we present its complete 3.91-Mbp genome sequence, generated via a combination of short-read sequencing (Illumina), long-read sequencing (PacBio), and manual finishing.

## GENOME ANNOUNCEMENT

Acinetobacter baumannii isolate A1 was isolated in 1982 at the Nottingham University Hospital in the United Kingdom (1) and is one of the earliest multiple-antibiotic-resistant isolates available in current collections. It has been recorded as being resistant to several of the antibiotics used therapeutically at the time, namely, sulfonamides, tetracycline, and gentamicin (2). A1 is also resistant to streptomycin and spectinomycin.

Whole-genomic DNA was sequenced on Illumina HiSeq at the Wellcome Trust Sanger Institute, generating 3,120,038 paired-end reads that were 100 bp in length, with a mean insert size of 275 bp. The reads were assembled *de novo* using Velvet version 1.2.10 (3) and VelvetOptimiser version 2.2.5 (http://bioinformatics.net.au/software.velvetoptimiser.shtml). The contigs were joined with the sequences of amplicons from polymerase chain reactions and assembled using Sequencher (Gene Codes Corporation, USA), producing an 8,731-bp plasmid sequence and a finished chromosomal sequence, except for the gene encoding the large repetitive protein known as the biofilm-associated protein. In order to resolve this complex repeat region, DNA was subjected to sequencing on 2 PacBio single-molecule real-time (SMRT) cells (chemistry version C2-P4) at DNA Link (South Korea). A total of 106,837 reads were obtained, with an average length of 8,033 bp and average quality of 0.82. The PacBio reads were assembled *de novo* using SMRT Analysis using the HGAP.3 algorithm with default parameters. This resulted in a plasmid sequence and a single contiguous chromosome sequence, with the repeat protein assembled completely. A comparison with the manually finished sequence indicated that apart from the repeat region, the only discrepancies between the assemblies were (i) a short sequence belonging to a repeated phage region that had been manually finished but was missing from the end of the PacBio sequence, (ii) 20 single-base differences, and (iii) 9 insertion/deletion variants of 1 to 3 bp. We therefore extracted the assembled repeat region from the PacBio sequence and inserted it at the corresponding location in the manually finished sequence, and we confirmed the correct base calls at the discrepant sites by manual inspection of the Illumina reads aligned to the assembly using Burrows-Wheeler Aligner (BWA) (4) or by capillary sequencing. The final assembly consists of 3,909,008 bases. The protein-coding, rRNA, and tRNA gene sequences were annotated using Prokka (5), and the resistance and polysaccharide loci (outlined below) and plasmid were annotated manually.

The genome sequence confirms that A1 is a member of global clone 1 (GC1; sequence type 1 [ST1] in the Institut Pasteur multilocus sequence type [MLST] scheme [6] and ST109 in the Oxford scheme [7]), one of the resistant clones found on all inhabited continents. It carries the KL1 capsule locus and the OCL1 outer core locus (8). Its antibiotic resistance is due to the presence of the *sul1*, *tetA(A)*, and *aacC1* genes, together with the *aadA1* gene in AbaR24, a genomic resistance island derived from AbaR0 (9) via an IS*26*-mediated deletion of a 10,876-bp segment that included the *aphA1b* and *bla*_TEM_ genes. There are no copies of the insertion sequence ISAba*1*. The plasmid pA1 is cryptic. The genome sequence of A1 will underpin studies of the evolution of the AbaR-carrying branch of the GC1.

### Nucleotide sequence accession numbers. 

The complete genome sequence has been deposited in DDBJ/ENA/GenBank under the accession numbers CP010781 (chromosome) and CP010782 (plasmid). The versions described in this paper are the first versions, CP010781.1 and CP010782.2, respectively.
